# MT3 melatonin binding site, MT1 and MT2 melatonin receptors are present in oocyte, but only MT1 is present in bovine blastocyst produced in vitro

**DOI:** 10.1186/1477-7827-10-103

**Published:** 2012-12-03

**Authors:** Rafael V Sampaio, Dhúllia Stefanne B Conceição, Moysés S Miranda, Lucia de Fatima S Sampaio, Otávio Mitio Ohashi

**Affiliations:** 1 Lab. Fertilização in vitro, Instituto de Ciências Biológicas, Universidade Federal do Pará, Federal do Pará, Brazil; 2 Lab. Bioquímica do Desenvolvimento do Sistema Nervoso, Instituto de Ciências Biológicas, Universidade Federal do Pará, Rua Augusto Corrêa 1. CEP: 66075-900, Belém, PA, Brazil; 3 Faculty of Animal Science and Food Engineering, University of São Paulo, Pirassununga, SP, Brazil

**Keywords:** Melatonin, Antioxidant, MT1 melatonin receptor, MT2 melatonin receptor, NQO2, Mechanisms of development

## Abstract

**Background:**

Melatonin inclusion into in vitro oocyte maturation (IVM) protocols has been suggested because it possesses a powerful free radical scavenger capability that improves the quality of the oocyte used in in vitro embryo production (IVP). The aim of our study was to investigate the presence of melatonin membrane receptors (MT1and MT2) and MT3, which is the melatonin binding site of NQO2 enzyme, in both oocytes and hatched blastocysts to consider an additional subcellular mechanism responsible for the effects of melatonin on IVP.

**Methods:**

The presence of the high affinity melatonin receptors was investigated through an autoradiographic binding assay, using the non-permeable ligand [^125^I]-iodomelatonin (17 pM) in embryos. The kind of melatonin site was investigated in oocytes and embryos by immunocytochemistry. In vitro fertilized bovine embryos produced from in vitro maturated oocytes supplemented with melatonin (0.0001 to 1000 nM) were analysed to determine their cleavage and blastocyst formation rates.

**Results:**

The [^125^I]-iodomelatonin (17 pM) binding in blastocysts was blocked by pre-incubation with melatonin (30000 nM), showing the presence of the high affinity melatonin receptors. MT1, MT2 and NQO2 immunoreactivity was observed in oocytes. MT1 immunoreactivity was observed in hatched blastocysts, however MT2 and NQO2 were not observed in this embryonic stage. Melatonin (pM) triggered significant difference in both cleavage and blastocysts formation rates.

**Conclusions:**

The high affinity MT1 melatonin receptor must be taking part in IVM events; furthermore it is the first melatonin receptor to appear during bovine embryo development in vitro.

## Background

Environmental lighting controls temporal changes for organisms that live on earth. In mammals, the major biological clock is located in the suprachiasmatic nucleus (SCN). This major clock drives other minor biological clocks that are located in the peripheral tissues. In the dark phase of the circadian rhythm, the SCN signals the pineal gland to release indoleamine melatonin (N-acetyl-5 methoxytriptamine). During pregnancy, maternal melatonin crosses the placenta, signalling the day length for the fetus, and modulates fetal development [1-4].

In development, melatonin acts by means of the MT1 and MT2 membrane receptors (Mel1a and Mel1b for avian), mainly by inhibiting the adenylate cyclase enzyme [3]. Due to the lipophilic nature of melatonin, it is able to function by binding to receptor in the nucleus [5], a cytosolic binding site the calmodulin protein [6] and also by modulating the cytosolic detoxifying enzyme NRH: Quinone Oxireductase 2 (NQO2). It does so through the MT3 melatonin binding site, which was showed to be NQO2 [7]. Furthermore, it has been observed that melatonin modulates the myeloperoxidase enzyme [8]. However, the most primitive mechanism of melatonin action is the direct scavenger function on reactive oxygen species (ROS), such as hydrogen peroxide (H_2_O_2_) and the super-oxide anion (O_2_^-^). This neurohormone also has an indirect antioxidant role thought to be triggered by membrane receptors and binding sites, mainly resulting in the up regulation of the antioxidant enzymes (glutathione peroxidase, glutathione reductase, gamma-glutamyl cisteine synthetase and glucose 6-phosphate deydrogenase) and the down regulation of the oxidant enzymes (NO synthase and lipoxygenases) [9,10].

Furthermore, melatonin modulates mammalian reproduction [11,12]. In the ovary, this hormone protects and stimulates folliculogenesis [13]. Its synthesis takes place in both cumulus cells and oocytes, where it reaches high levels. In fact, ovarian follicular fluid contains melatonin in higher concentrations than in plasma. At these concentrations, melatonin has a protective action by acting as a direct free radical scavenger [14,15]. Additionally, several effects of melatonin on the ovary are triggered by its membrane receptors that are also underlying its effects on photoperiodism, and on other events related to reproduction [16-21].

The direct free radical scavenger action of melatonin is very useful to the development of an embryo produced in vitro. It is important to note that species-specific concentration of the melatonin included in oocyte in vitro maturation (IVM) protocols increases the production of in vitro fertilized embryos in mice [22,23], pig [24] and buffalo [25]. The addition of melatonin is very useful as a sperm cryoprotective agent as well [26].

The addition of melatonin in IVM medium is justified because in vitro production (IVP) is strongly influenced by events before in vitro fertilization (IVF), particularly during IVM [27,28]. The maturation process is where the oocyte acquires “competence” to assure proper zygotic development until the blastocyst stage [29]. It has been showed that melatonin has a long-term effect on embryos, which is observed in cleavage and blastocysts formation rates. The efficacy of melatonin depends on culture conditions, such as O_2_ tension [30]. This dependence of the O_2_ tension further suggests that melatonin influences in vitro embryogenesis by acting as a radical free scavenger. Others constituents of the IVM culture medium (i.e. FSH and LH) [31] can also influence the melatonin efficacy on oocyte maturation as well. Beside animal reproduction, melatonin has been used in human assisted reproductive technologies. To increase fertilization and pregnancy rates for female infertility due to poor oocyte quality, oocytes are treated with melatonin. This results from melatonin reduction of the toxic products caused by oxidative stress during oocyte maturation [32].

The aim of the study was to evaluate the effects of melatonin on bovine oocyte IVM by measuring the cleavage rates and blastocysts formation rates, as well as investigating the presence of the NQO2 enzyme, and MT1 and MT2 melatonin membrane receptors in bovine oocytes and blastocysts.

## Methods

### In vitro embryo production (IVP)

Institutional approval was received for the work with embryos in this study (94/2010-ICB). The IVP was performed as previously described [33]. Briefly, bovine ovaries were obtained from a slaughterhouse and transported to the laboratory in saline solution (0.9% NaCl). Cumulus-oocytes complexes (COCs) were recovered from small antral follicles (2–8 mm) by follicular aspiration. Only COCs with at least three compact cumulus cell layers and homogeneous cytoplasm [34] were selected for IVM. Groups of 15 COCs were cultured in a 100 μl droplet of TCM-199 supplemented with sodium bicarbonate, 10% of FBS (Gibco BRL, Grand Island, NY, USA), 11 mg/ml of pyruvate, 50 μg/ml of gentamicin, 0.5 μg/ml of FSH (Folltropin, Bioniche Animal Health Belleville, Ont., Canada), and 5.0 μg/ml of LH (Lutropin, Bioniche Animal Health Belleville, Ont., Canada). The dishes were covered with mineral oil and incubated for 24 h in a humidified atmosphere of 5% CO2 in air at 38.5°C. Frozen-thawed bull semen (*Bos taurus*) was prepared for IVF by density gradient centrifugation in Percoll. Sperm cells were counted in Neubauer chamber and then added to IVF droplets (2 × 10^6^ spermatozoa/ml). Matured oocytes were washed, transferred to IVF droplets (80 μl of TALP-FERT medium supplemented with heparin, penicillin, hypotaurine, epinephrine and BSA) and incubated in an atmosphere of 5% CO2 in air at 38.5°C. After 24 h of IVF, cumulus cells that were still surrounding the fertilized oocytes were mechanically removed by successive pipetting. Groups of 20 presumptive zygotes were washed and transferred to 100 μl droplets of Synthetic Oviduct Fluid [35] supplemented with 2.5% (v/v) FBS. Embryo development was supported by a co-culture with a granulosa cell monolayer in an atmosphere of 5% CO2 in air at 38.5°C for 7 days.

### Melatonin treatment during IVM and embryonic analyses

After selection, pools of 15 COCs were randomly distributed in seven groups and incubated in droplets of IVM medium without melatonin (control) or supplemented with 0.1 pM, 1 pM, 0.01 nM, 0.1 nM, 1 nM, and 1 μM of melatonin (Sigma Co., St. Louis, MO, USA). Melatonin was diluted in DMSO plus medium (V/V), using a stock solution of 15 mM, which was diluted up to 0.1 pM using only IVM medium. The effect of melatonin during IVM was evaluated further in bovine embryo development after IVF. Cleavage was recorded at day 2 and blastocyst formation was evaluated at day 7 of IVC (IVF was set as Day 0). Obtained blastocysts were fixed in a paraformaldehyde solution (3.7%) for 15 minutes and then stained with 5 μg/ml of Hoechst 33342 (Sigma Co., St. Louis, MO, USA) for 10 minutes and visualized by fluorescence microscopy (Nikon Eclipse TE300). Nuclei were counted from pictures taken from individual blastocysts. The higher dose of DMSO used was without effect, comparing with controls non-melatonin treated.

### Autoradiography binding assay

Two pools of six day 7 bovine blastocysts in vitro-produced from oocytes maturated without melatonin were incubated in a serum free medium containing the melatonin membrane receptors agonist [^125^I]-iodomelatonin (NEN™ Life Sciences Products, Inc. Boston, MA, USA) (17 pM) for 1 h. The pool used for non-specific binding was incubated in the presence of melatonin (30000 nM) for 30 min. After incubation, the embryos were washed with phosphate buffer solution (PBS, pH = 7.4), placed in slides and dried. The slides were exposed to [^125^I] sensitive film (Kodak insight, Super Poly-Soft 1238054, New York, USA) for two weeks, and then the film was developed, fixed (Kodak developer CAT 8610248 and Kodak Fixer CAT 8610248, New York, USA), rinsed and dried. The image was captured by scanning (HP Scanjet 2400) and then analysed using Image J (NIH, Bethesda, MD, USA).

### Immunocytochemistry

The melatonin receptors (MT1 and MT2) and NQO2 enzyme were investigated in oocytes maturated in absence of the melatonin and day 7 blastocysts. Primary (NQ02 (N-15): sc-18574; MEL-1B-R (T-18): sc-13177; MEL-1A-R (R-18): sc-13186) and secondary (Alexa 594-conjugated anti-goat IgG-CFL 594) antibodies were purchased from Santa Cruz Biotechnology (CA, USA). The embryos were washed in phosphate buffer saline (PBS), fixed in 4% paraformaldehyde for 15 min at room temperature and then rinsed in PBS. Next, embryos were incubated in 1% bovine serum albumin (BSA) for 15 min at room temperature (blocking) and then incubated with primary antibodies diluted (1:100) in PBS overnight at 4°C. Embryos were extensively washed in PBS and then incubated for 45 min at room temperature with 1:200 of Alexa 594-conjugated anti-goat antibody. Cell nuclei were stained with 5 μg/ml of Hoechst 33342 in PBS for 15 min at room temperature. Finally, embryos were mounted on each microscope slide and analyzed on a fluorescence microscope (Nikon Eclipse TE 300, Nikon Instruments Inc., Tokyo, Japan).

### Data analyses

Statistical analyses were done using the BioEstat 5.0 software. A value of p < 0.05 was considered to be significant. Data are presented as the mean ± standard error of the mean (S.E.M.).

## Results

### Autoradiography binding assay

**Figure 1:**
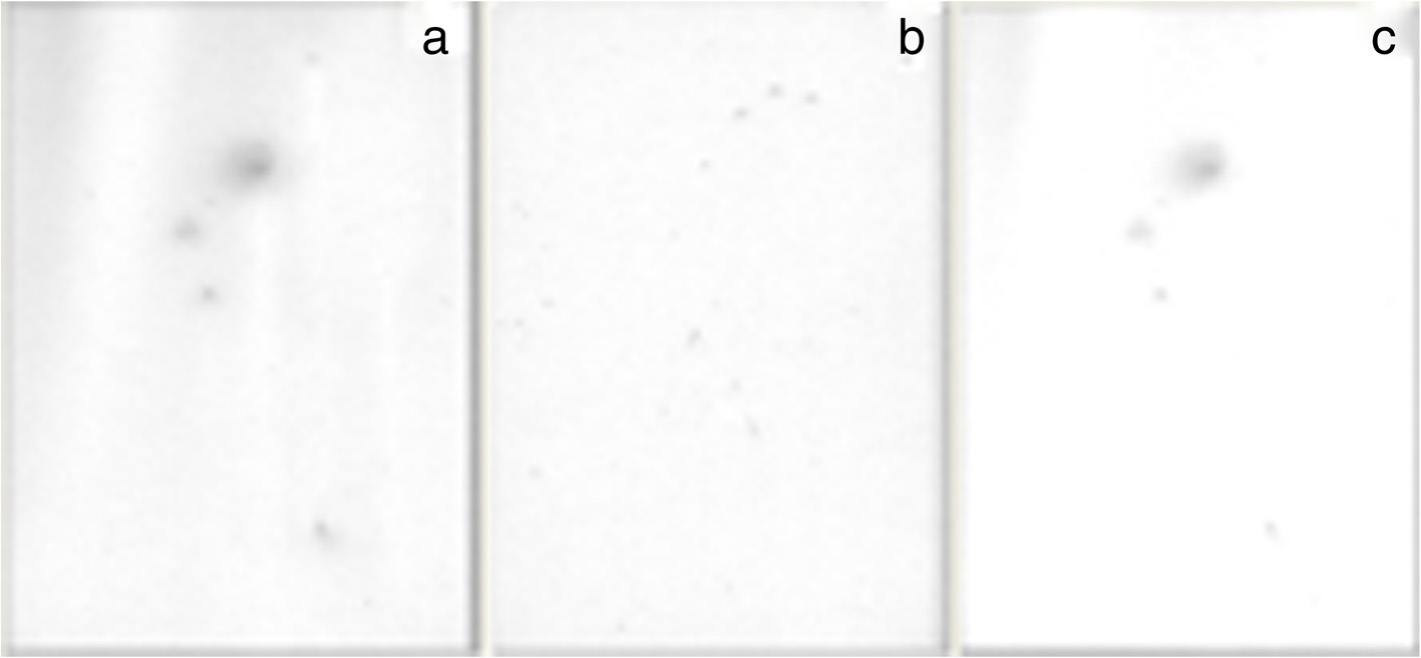
**High affinity [**^**125**^**I]-iodomelatonin (17 pM) binding showed by autoradiography in blastocysts.** Autoradiograms of the total (**a**), non-specific (30000 nM melatonin presence) (**b**), and specific (**c**) [^125^I]-iodomelatonin (17 pM) binding in seven days old bovine embryos that were produced without melatonin inclusion in oocyte maturation protocol. The autoradiograms are actual size (240 μm, embryo diameter).

We began by investigating the presence of melatonin receptors in bovine embryos cultured for 7 days. Data from autoradiography binding assay showed specific binding to [^125^I]-iodomelatonin (17 pM) (Figure 1c) that was blocked by pre-incubation with 30000 nM melatonin (non-specific binding) (Figure 1b). The total binding (specific binding plus non-specific binding), is also showed (Figure 1a).

### MT1 and MT2 receptors and NQO2 enzyme immunocytochemistry in bovine blastocyst

**Figure 2:**
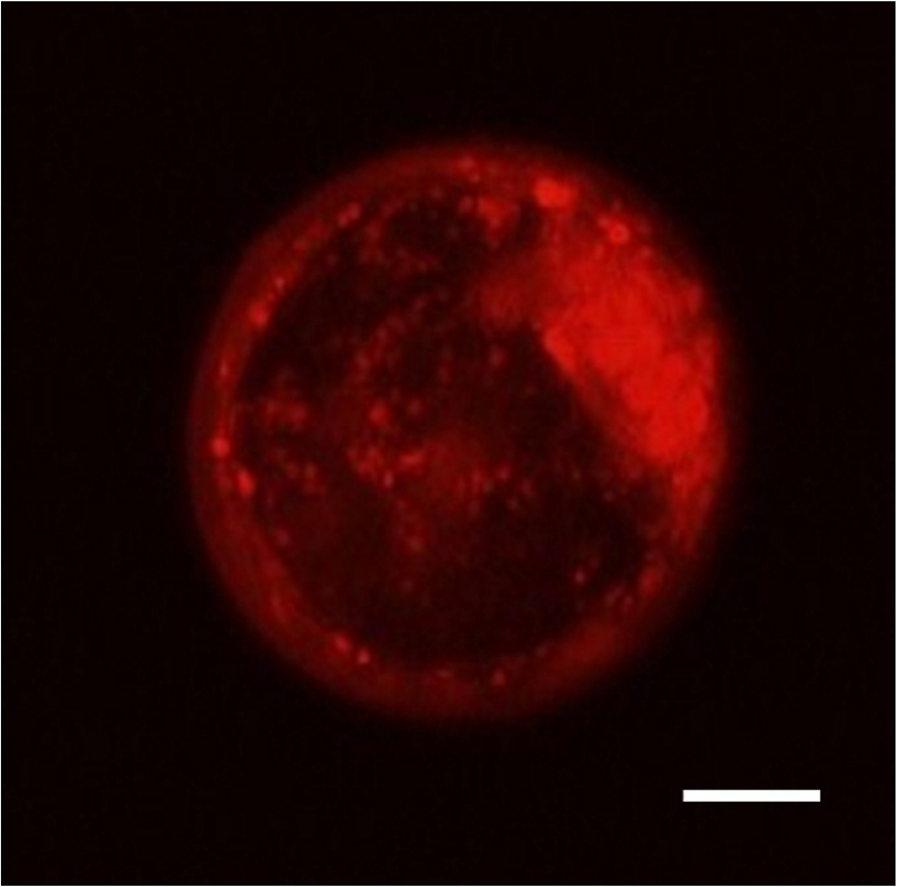
**Distribution of the melatonin receptor MT1 in bovine blastocyst.** Immunocytochemistry staining day 7 embryo produced without melatonin inclusion in oocyte maturation protocol. Positive staining for MT1 (red) is present throughout the blastocyst. The embryoblast cells show stronger staining for MT1 than the trophoblastic cells (Scale bar, 50 μm).

The high affinity melatonin receptor found by autoradiography in blastocysts was investigated by immunocytochemistry. Immunoreactivity neither observed for the melatonin membrane receptor MT2 nor for the cytosolic melatonin binding site NQO2. However, a strong immunoreactivity was observed for the melatonin receptor MT1 (Figure 2).

### Melatonin receptors MT1, MT2 and NQO2 enzyme immunoreactivity in bovine oocytes

**Figure 3:**
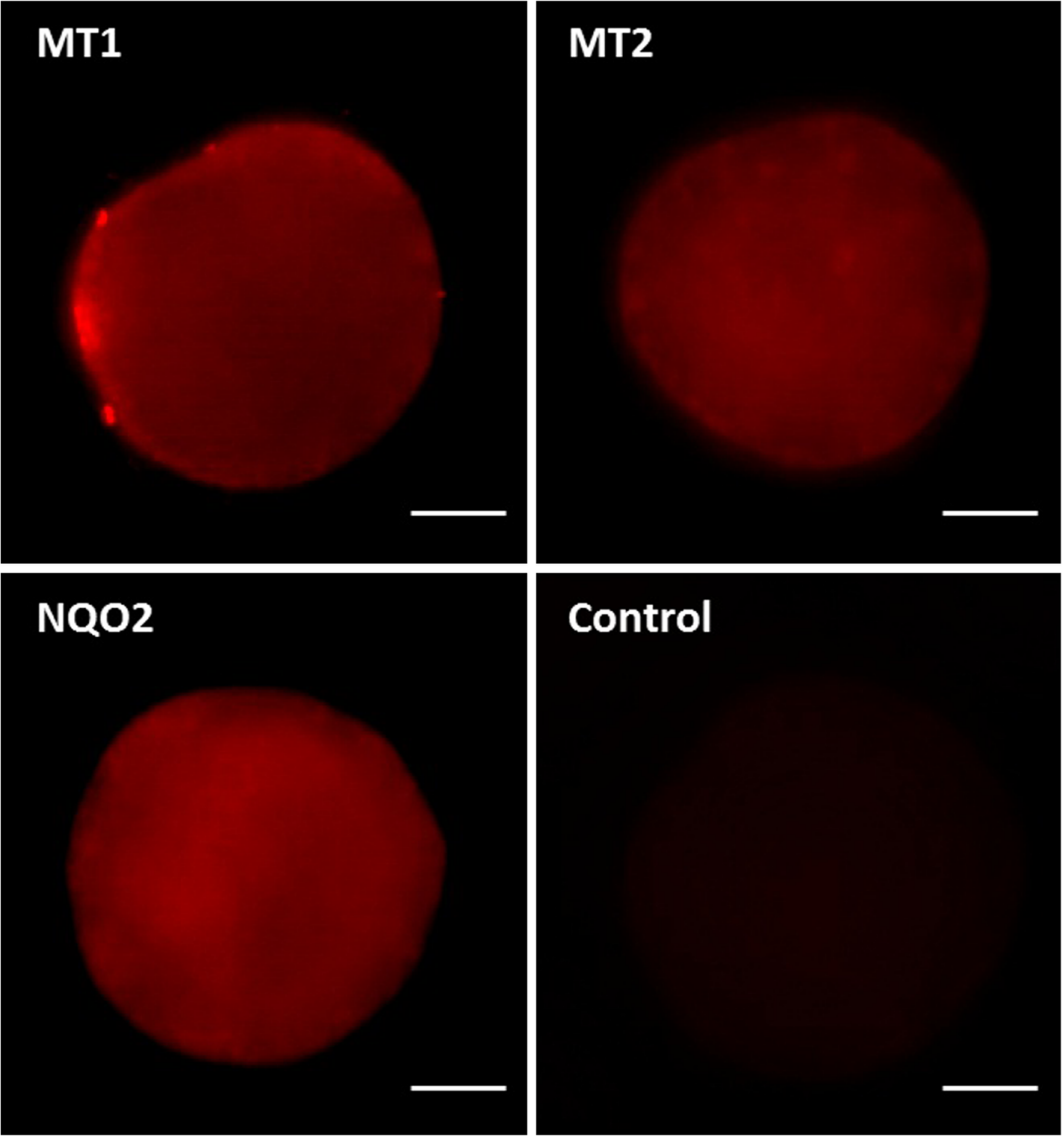
**Immunoreactivity patterns of melatonin receptor MT1, MT2 and NQO2 in bovine oocyte.** MT1, MT2 melatonin receptors and NQO2 are expressed by the matured oocyte. The upper-left panel shows localized intense MT1 staining suggesting that MT1 is expressed by polar bodies. The upper-right panel shows a cluster-like distribution of MT2 throughout the matured oocyte. The bottom-left panel shows a diffuse expression of NQO2 enzyme, and the bottom-right shows the negative control. (Scale bar, 30 μm).

High affinity melatonin membrane receptors and the NQO2 enzyme were investigated by immunocytochemistry in mature oocytes. It was observed immunoreactivity for MT1, MT2 and NQO2 enzyme. The pattern of immunoreactivity was different, as showed in the figure (Figure 3 a, b and c).

### Melatonin effects on in vitro bovine embryogenesis

The effects of melatonin (0.1 pM to 1000 nM) on bovine oocyte IVM and embryo development were analysed at day 2 (cleavage rate) and at day 7 (blastocyst yields). Melatonin improved the quality of bovine oocytes maturate in vitro, resulting in an increase in the cleavage rate and in significant blastocyst formation rate with an optimum concentration of 1 pM. However, the cleavage rate of embryos from oocytes treated with melatonin presented a gradual decline in response to increased melatonin concentration beyond 1 pM. Similar results were obtained when melatonin-treated blastocysts development was examined. The improvements of melatonin to blastocyst development decreased in a similar manner as observed for embryo cleavage over the same concentration range (Table 1). The best melatonin concentration to trigger an increase in total cell number of bovine blastocyst also was 1 pM, however, no inhibitory effects were observed with concentrations up to 1000 nM (data not showed).

**Table 1:** Embryo production

**Group**	**N**	**Cleavage rates (%)**	**Blastocyst yield (%)**
*Control*	75	52.9 ± 2.8 ^a^	30.9 ± 3.9^a^
*0.1 pM*	60	57.3 ± 6.6 ^a^	32.0 ± 6.6^b^
*1 pM*	75	62.1 ± 5.0 ^a^	43.7 ± 2.3^c^
*0,01nM*	75	52.7 ± 1.8 ^a^	34.2 ± 3.6^a, b^
*0,1nM*	75	39.4 ± 4.0 ^b^	19.8 ± 3.7^a, b^
*1nM*	55	40.4 ± 8.7 ^b^	30.0 ± 3.6^b^
*1 μM*	75	34.4 ± 3.5 ^b^	25.5 ± 1.3^d^

^a,b,c,d^ Values in the same column with different superscripts differ significantly (P < 0.05). N, total number of oocytes.

## Discussion

In this study we showed that a pM concentration range of melatonin supplementation in IVM medium functionally increases cleavage and blastocysts formation rates. However, while increasing the melatonin concentration up to a μM range, the positive effects of melatonin decreased in comparison with the values of the control group. This profile is in accordance with the presence of the melatonin targets that bind with different affinities in oocytes, and the most effective concentration range is related to the MT1 melatonin receptor. In agreement with this pharmacological profile, MT1, MT2 and NQO2 immunoreactivity were visualized in oocytes for the first time in this study. Interestingly, in blastocysts, the autoradiographic binding assay showed a high affinity site, and immunocytochemistry assays only showed the presence of the MT1 melatonin receptor. Since the embryonic genome is activated at the 8-cell stage in bovine embryos, there is a complete absence of maternal genetic inheritance in day 7 blastocysts [36]. Thus, this study shows the novel findings that the MT1 melatonin receptor is the first to be transcribed in a mammalian embryo.

Herein, we also showed for the first time the presence of the cytosolic NQO2 enzyme in oocyte. This detoxifying enzyme is thought to produce free radicals when activated by xenobiotics, such as menadione and is inhibited by high concentrations of melatonin [37], as found in the bovine follicular fluid [15]. Probably its inhibition by melatonin takes part in general defense mechanisms of the oocyte. A recent paper showed a melatonin protective action against HOCl, preventing oxidative damage to the metaphase-II oocyte spindle in vitro, when it was added in IVM medium. This effect was via the inhibition of the myeloperoxidase enzyme [8], being more one study showing that melatonin protection occurs by a myriad of mechanisms.

Despite powerful scavenger actions being considered the pivotal mechanism underlying melatonin protection in different cells [9,38], melatonin membrane receptors are also important as they have a role in spermatozoa protection in vitro [39]. Furthermore, it was recently shown that the melatonin membrane receptors mRNAs are present in bovine oocytes as well as bovine cumulus cells and positively affect cytoplasmic and nuclear maturation [40]. Regarding melatonin mechanism of the action, in general, MT1 and MT2 signaling pathways are not restricted to the Gi protein subunit, resulting in an inhibition of the adenylate cyclase enzyme. MT1 potentiates phosphoinositide turnover, when it is stimulated by another receptor, while MT2 can act by coupling with the Gq protein subunit. This stimulates phosphoinositide turnover and, by others means, inhibits the guanylate cyclase enzyme. Therefore, MT1 and MT2 signaling pathways can induce PKC activation by Ca^2+^ waves, in parallel with the inhibition of the cAMP pathways [41,42].

The mechanism of the oocyte self-maintaining in arrest is poorly understood, however, a review about its control shows that bovine oocyte maturation is modulated by inhibition of both cAMP and cGMP levels as well as by the energy sensor AMPK, which is activated by AMP. In addition, there are studies showing the importance of the Ca^2+^ and PKC to bovine oocyte maturation [43]. MT1 and MT2 inhibit the adenylate cyclase enzyme activity and are able to activate PKC, which consequently has proven to improve nuclear maturation and the quality of IVP blastocysts. The pM concentration range of melatonin observed to affect the cleavage and blastocysts formation rates is suggestive of the melatonin action on the MT1 receptor [41]. Interestingly, the MT1 melatonin receptor was characterised by autoradiography and immunocytochemistry in early day seven blastocysts, demanding further investigations as to its constitutive activity at this embryonic age, which will largely enhance the understanding about melatonin influence on bovine embryonic development.

## Conclusions

Along with the clear immunoreactivity to MT1, MT2 and NQO2 in oocytes, the addition of a melatonin pM concentration increased cleavage and blastocysts formation rates. We suggest this is due to the effects of the MT1 receptor. We suggest that this hormone does not only have free radical scavenger action, but it also has effects on oocyte maturation by a myriad of mechanisms, including membrane receptor activation.

## Abbreviations

AMPK: Adenosine monophosphate-activated protein kinase; IVF: In vitro fertilization; IVM: In vitro maturation; IVP: In vitro production; NQO2: NRH:quinone oxidoreductase 2; MT3: Melatonin binding site; PKC: Calcium-activated kinase; SCN: Suprachiasmatic nucleus.

## Competing interests

The authors declare that they have no competing interests.

## Authors’ contributions

RVS carried out the autoradiographic binding and IVP assays, and helped to draft the manuscript. SDBC carried out IVP and immunoassays. MSM participated in coordination, immunoassays, performed the statistical analyses and helped to draft the manuscript. OMO participated in its design and coordination and critically review the manuscript. LFSS conceived of the study, participated in its design and coordination, and draft the manuscript. All authors read and approved the final manuscript.
